# Nutritional status of displaced children including unaccompanied minors on Lesvos, Greece

**DOI:** 10.1016/j.jmh.2025.100393

**Published:** 2025-12-27

**Authors:** H. Benjeddi, M.P. Gruppen, S.A. Post, A.D. Groenewegen, E.B.K. Egen, E. Samiotaki Logotheti, Z. Livaditou, W.P. Voskuijl, A.M. Tutu-van Furth, M. Boele van Hensbroek, A. Terzidis, M. van der Kuip

**Affiliations:** a Amsterdam UMC, location University of Amsterdam, Amsterdam Institute for Global Child Health, Emma Children’s Hospital, Meibergdreef 9, Amsterdam, Netherlands; b Department of Pediatric Infectious Diseases, Rheumatology and Immunology, Amsterdam University Medical Center, Emma Children’s Hospital, Meibergdreef 9, 1105 AZ Amsterdam, Netherlands; c School of Medicine, National and Kapodistrian University of Athens, Greece; d Department of Pediatric Infectious Diseases, Rheumatology and Immunology, Amsterdam University Medical Center, Emma Children’s Hospital, Amsterdam Infection & Immunity Institute, Vrije Universiteit Amsterdam, De Boelelaan 1117, 1081 HV, Amsterdam, Netherlands

**Keywords:** Refugee camps, Displaced children, Refugee, Asylum seekers, Nutritional status, Growth status, Unaccompanied minors, UAMs

## Abstract

**Introduction:**

The number of displaced people worldwide has reached an unprecedented 120 million, of whom 40% are children. There is limited knowledge about the health of children in refugee camps, particularly regarding their nutritional status. This study examines the nutritional status of 304 displaced children (0–18 years) in Closed Controlled Access Centre (CCAC) Mavrovouni, Lesvos, Greece.

**Methods:**

An observational study was conducted between February and August 2023 at CCAC Mavrovouni. Data collection included demographics, dietary details, health and nutritional status. The primary outcome was the prevalence of wasting, stunting and overweight using World Health Organization (WHO) criteria. We also conducted subgroup analyses for unaccompanied minors (UAMs) separately. Secondary outcomes of our study included the role of breastfeeding and other factors that potentially affect nutritional status.

**Results:**

The overall prevalence of stunting among minors in CCAC at baseline was 11%. 10% of children under five were wasted. Of all children, 5% underweight and 13% were overweight. Nutritional status does not change during their stay in the camp: wasting (improvement in Z-score by 0.2, 95% CI -0.5–0.1), stunting (decrease in Z-score by 0.07, 95% CI -0.2–0.3). There was a significantly higher prevalence of stunting in the UAM sub-group (31%, *p* < 0.01). No association was found between breastfeeding and weight-for-height Z-scores under two years old (Z-score difference of 1.3, *p* = 1.18), but there was in the larger age group up to five (Z-score difference of 0.82, *p* < 0.01).

**Conclusions:**

Our results show that poor nutritional status is prevalent amongst displaced children on Lesvos, highlighting their vulnerability. Our results underline the compromised health and vulnerability of UAMs, with nearly one third of this group being stunted.. The unexpected prevalence of overweight highlights complex nutritional challenges.

## Introduction

The global increase in forced displacement has reached unprecedented heights, with projections indicating 120 million people in April 2024 (United Nations High Commissioner for Refugees, 2024), of whom 40 % are children. Of the total of 43.4 M refugees, 73 % originate from 5 countries: Afghanistan (6.4 M), Syria (6.4 M), Venezuela (6.1 M), Ukraine (6 M) and South-Sudan (1.5 M) (United Nations High Commissioner for Refugees, 2024). Conflict in the Gaza Strip has recently caused a surge in the number of Palestinian refugees (6 M). While Europe has many temporary settlements, accurately determining the exact number of refugees in the region is complicated, due to differing definitions and reporting methods (Katz, 2016). Despite the refugee agreement between the European Union and Turkey in 2016 (International Rescue Committee, 2023), the Eastern Mediterranean route through Greece remains an important gateway for refugees entering Europe. The Eastern Mediterranean route is a path used by refugees from the Middle East, North Africa, and South Asia to reach Europe. They typically travel through Turkey and cross the Aegean Sea to Greece, often in unsafe boats (Dilger, 2025) . Greece hosts over 30 refugee accommodation facilities and 6 so-called Reception and Identification Centers (Krithari, 2024) . The island of Lesvos serves as the primary entry point and hosts one of Europe’s largest refugee camps, known as Closed Controlled Access Center (CCAC) Mavrovouni and also referred to as Kara Tepe camp. It was established in September 2020 as an emergency response after the Moria refugee camp was destroyed in a fire (Smith, 2020). Its population fluctuates between 1000 and 5000 individuals, reflecting a diverse demographic profile based on countries of origin and the current humanitarian crises (EuroRelief, 2024). Numerous governmental and non-governmental organizations are instrumental in providing urgent healthcare services, especially for children in CCAC Mavrovouni. To avoid confusion regarding legal terminology related to refugees and migrants, we use the term "displaced children" to describe all children in the camp.

Children face emotional distress on various levels, with the loss of their home and the perilous journey being the most significant factors (Foka et al., 2021). There are premigration, peri‑migration and postmigration factors impacting their health (Hazer et al., 2023). Before reaching the shores of Greece from Turkey, many have often attempted to cross the sea multiple times, facing intimidating circumstances. A specific group of these children, known as unaccompanied minors (UAMs), arrive without parents for two main reasons. Some are orphans who lost their parents during conflicts in their home countries, while others travel alone with plans to reunite with their families later on.

Little has been studied on the topic of health status of displaced children upon arrival at refugee camps, both globally and specifically at CCAC Mavrovouni. Increased exposure to infections, limited access to adequate health care (e.g., vaccinations), and poor hygiene can lead to comprised health (Altare et al., 2019). Nutritional status is an established factor that reflects overall health and socio-economic status in children (Solar, 2007). Forced migration can create and exacerbate compromised nutritional status: the disruption of familiar environments and access to food resources can lead to acute malnutrition in the short term, while long-term displacement often results in chronic undernutrition, in part due to pre-departure stressors (Lancet Editorial, 2023).

In order to understand what is scientifically known about the nutritional status of displaced children living in refugee camps in Europe specifically, we conducted a systematic review and meta-analysis, that was recently published (Benjeddi et al., 2023). In that review by our group, which encompassed 12 studies and included 7009 children from 14 refugee camps in and around Europe, we found a pooled prevalence of stunting of 16 % and wasting of 4 %. Our review also underscored that there is a lack of research on how nutritional status of refugee children may evolve over time, as well as the importance of including potentially associated variables, such as the practice of breastfeeding and being a UAM, in surveys and analyses (United Nations, 2005; Maioli et al., 2021).^,^ Only one study describes the nutritional status of UAMs in camp Moria on Lesbos, showing a prevalence of 24 % underweight among 63 UAMs that were included (Bydzovsky et al, 2021).

This study aimed to examine the nutritional status of children in CCAC Mavrovouni with an additional focus on UAMs. We hypothesized that study participants’ overall nutritional status would improve over time in CCAC Mavrovouni. Additionally, we hypothesized that UAMs would exhibit poorer nutritional status.

## Methods

Study design and participants: In this study, we conducted an observational study within CCAC Mavrovouni on the island of Lesvos, Greece, as well as within shelters on this island dedicated to care for UAMs. This research project formed part of the Mavrovouni Migrant Child Health Project, an initiative that aims to investigate the health status of children arriving at CCAC Mavrovouni and the effects of living in the camp on their physical and psychological health. This is a collaboration between the National and Kapodistrian University of Athens in Greece, the University of Amsterdam and the VU University Amsterdam in the Netherlands. Data were collected on working and weekend days, depending on the availability of interpreters. The data collection ran from 9 February 2023 to 14 August 2023. The research facilities were situated within the medical area of CCAC Mavrovouni, in addition to four distinct UAM shelter locations distributed across the island of Lesvos. See Appendix I for a map of CCAC Mavrovouni.

The primary outcomes were the prevalence of wasting and stunting determined by Z-scores for weight-for-height, height-for-age, and BMI as measured at one point in time during our six-month period of data-collection. Secondary outcomes included comparison of nutritional status between children with families and/or caregivers to UAMs. Other secondary outcomes were comparison of nutritional status between different age categories, malnutrition among breastfed children, the prevalence of malnutrition based on mid-upper arm circumference (MUAC) and the development of nutritional status at follow-up. Evident clinical signs of malnutrition were assessed as another secondary outcome.

Infants, children and adolescents aged 0 to 18 years, were screened for eligibility for inclusion. Age assessment was based on their official asylum documentation which was obtained from the reception and identification administration upon arrival to Lesvos. All children living in CCAC Mavrovouni were eligible for inclusion if they were accompanied by a parent and/or guardian. Informed consent was requested from a parent or legal guardian. For those aged 12 years or older, informed assent was used as a supplementary mandatory component of the enrollment process. UAMs were considered eligible for participation if they were residing in one of the designated shelters serviced by the largest NGO responsible for the care of UAMs in Greece. They facilitate accommodation, food, psychological care and education for UAMs on the island. In the present study, informed consent was obtained with approval from the public prosecutor. Additionally, individuals aged 12 years or older were also required to provide informed assent as a condition for inclusion in the study.

The camp management provided a list featuring potential study participants based on arrival date. Children and their families that had newly arrived were given priority so as to include children as early as possible during their stay in the camp. We aimed to do the baseline measurement within four weeks after arrival. Thereafter, the coordination of appointments was facilitated by a non-governmental organization (NGO) operating within the camp. Emphasis was placed on ensuring equal representation from different countries of origin. The UAMs only had one appointment due to logistical reasons.

In order to arrange follow-up assessments after 3 months, initial contact was established by placing telephone calls to a contact number provided during the first visit. When direct communication was not possible, inquiries were made to the camp management regarding the current location or status of the participants. If the subjects were still residing within the camp, efforts were made to arrange visits to their accommodations. However, if it was determined that they had already left the camp, they were classified as ‘lost to follow-up.’ Oral informed consent and assent were administratively provided.

*Ethics:* Ethical clearance for this research project was obtained from the Medical Ethics Committee of the Greek National Public Health Organization (EODY). This was provided in December 2022 under Reference No. 23,702/06–12–2022 (see Appendix II).

*Procedures:* A questionnaire addressing demographics, general health status, food sources and diet, including information on breastfeeding duration if applicable, was administered by trained research assistants. Interpreters from the camp translated the questions for participants if needed (see Appendix III). Research assistants comprised students in master’s-level research degree programs from four academic institutions in the Netherlands and Greece, who had backgrounds in medicine, global health, and disaster management and received preparatory training. The research coordinator conducted quality assessments of the data collection. Interpreters were recruited through collaboration with camp management as well as various NGOs that worked in the camp. They were assessed for eligibility and underwent training to ensure optimal interpretation. A total of six interpreters provided translation in Arabic, Farsi, Dari and Somali.

Anthropometric measurements were done by trained research assistants at the study clinics making use of a Seca 716 mechanical person scale and Seca 213 stand-alone length meter for weight and a height and a Seca 354 baby scale and Seca 207 baby length meter for the non-standing children (Seca manufactured in Hamburg, Germany). Mid-upper arm circumference (MUAC) was measured using standardized and internationally used methodology (Health Books International, 2024). There were no time restrictions for data inclusion.Children were screened for clinical signs of malnutrition, specifically: oedema, pallor, dry skin, dermatitis, dull nails, enlarged thyroid, cracked lips, bleeding gums, xerophtalmia, dull hair, excessive dental caries (CHAIN, 2018). A referral pathway to a Greek pediatrician in an academic hospital was established in case referral as deemed necessary.

*Data handling, definitions and statistical analysis:* The information was securely stored in a Castor cloud database (Castor EDC, 2022). Using a sample size of 300, a two-sided 95 % confidence interval for a single proportion extended 4.1 % from the observed proportion for an expected proportion of 0.16 based on our systematic review and meta-analysis (Benjeddi et al., 2023).. For each child, Z-scores were calculated using World Health Organization (WHO) Anthro tool (WHO Anthro, 2010) for children 0–59 months and WHO Anthro Plus tool for children aged 6–19 years of age (WHO AnthroPlus, 2009). This tool ensures correction for both gender as well as age. The calculated Z-scores were for length-for-age and weight-for-height. These Z-scores were analyzed using SPSS Statistics 28.0.1.1(IBM Corp, NY, 2022). MUAC Z-scores were obtained using the “Anthro” package in R (R Core Team, 2021). The WHO definitions of wasting (weight for height Z-score equal or smaller than – 2) and stunting (height-for-age Z-score equal or below −2) were used as parameters of malnutrition (WHO Nutrition Landscape Information System, 2025). The Mann-Whitney U test was used to assess statistical differences between non-normally distributed data. A paired T-test was used to assess the difference between two Z-scores in the same child over time. To assess the difference in stunting prevalence between two groups of children (accompanied children and UAMs), Fisher’s exact test was used. In all analyses, the significance level was 0.05. To explore potential correlations, a multivariable regression test was used to assess the relationships between the dependent variable of Z-score height-for-age at baseline and the following independent variables: the number of siblings, length of stay in the camp, and travel duration before camp arrival. Multicollinearity was assessed by examining the Variance Inflation Factor (VIF) in the multivariable regression. A VIF greater than 5 was considered indicative of potential issues requiring further investigation. However, no multicollinearity was detected among the included variables.

## Results

**Fig. 1:**
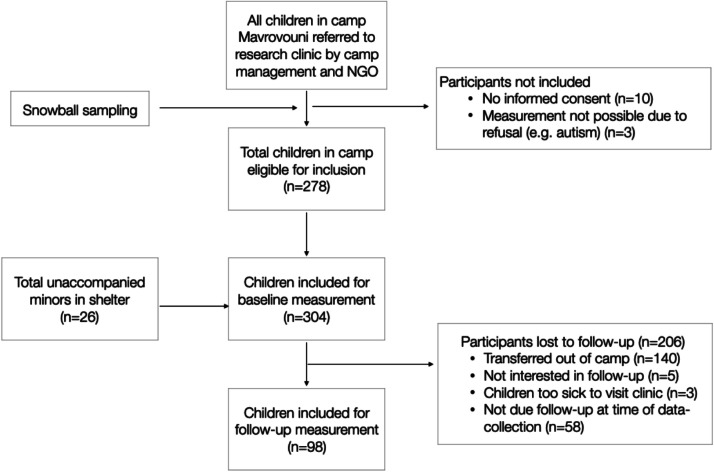
Flow diagram for participant inclusion.

We enrolled a total of 304 displaced children, aged 0–18 years, residing on the island of Lesvos. Fig. 1 shows the flow diagram for participant inclusion. Our sample represented 16 nationalities, with the majority (88 %) originating from five countries: Afghanistan, Somalia, Palestine, Yemen and Democratic Republic of Congo. Detailed baseline characteristics are presented in Table 1.

**Table 1:** Baseline characteristics.

**Demographics**			
	**0–5 years**	**6–18 years**	**Total**
Number	110	194	304
Age (median, yrs)	2 (IQR 1–3.8)	10.6 (IQR 7.7–14)	7.6 (IQR 3.4–12.2)
Gender: female ( %)	55	52	54
Country of origin based on asylum papers ( %) Afghanistan: Somalia: DR Congo: Palestine: Eritrea: Other:	55 11 8 7 6 14	60 10 9 7 4 11	58 10 8 6 5 12
Siblings ( %) No siblings: 1 sibling: 2 siblings: 3 or more siblings:	27 23 28 22	3 19 29 49	12 21 29 19
Chronic disease 1 ( %)	10	12	12
Vaccinated ( %)	90	95	93
Currently breastfed ( %)	40	0	14
**Travel information**			
	**0–5 years**	**6–18 years**	**Total**
Travel duration before arrival (median and range in mo)	8 0–72	7 0–108	7 0–108
Months in CCAC Mavrovouni (median and range in mo)	5 0–125	5 0–66	5 0–125
Resided in refugee camp before ( %)	9	19	16
UAMs	1 child (1 %)	25 children (13 %)	26 (9 %)

1 Chronic diseases that were reported include skin conditions, epilepsy, autism, anemia, solitary kidney and developmental delay.

Table 2 shows the prevalence of malnutrition among different age groups. On enrollment, the 0–5 years old age group had the greatest prevalence of stunting. Longitudinally, there were no statistically significant changes in wasting (increase in Z 0.2, 95 % CI −0.5–0.1) or stunting (decrease in Z 0.07, 95 % CI −0.2–0.3). We found that, upon arrival, 19 % of children in the 6–18 year age group were overweight, which decreased over time to 2 %. MUAC scores, collected for 83 children under 5 years of age, showed a median Z-score of 0.66. Based on MUAC scores, only one child was at risk of malnutrition and no children showed moderate or severe malnutrition.

**Table 2:** Percentages of malnutrition at baseline subdivided by age group including confidence intervals.

	**Stunting**^1^**(N, %, 95 % CI)**	**Wasting**^2^**(N, %, 95 % CI)**	**Underweight**^3^**(N, %, 95 % CI)**	**Overweight**^4^**(N, %, 95 % CI)**
*0–5 years (N=106)*	14 (13 %, 7–21 %)	11 (10 %, 5–18 %)	3 (3 %, 1–8 %)	4 (4 %, 1–9 %)
*6–18 years (N=198)*	18 (9 %, 5–13 %)	n/a	11 (6 %, 2–9 %)	37 (19 %, 13–24 %)
**Total (*N*=304)**	**32 (11 %, 7–14 %)**	11 (10 %, 5–16 %)*	**14 (5 %, 2–7 %)**	**41 (13 %, 10–17 %)**

1 height-for-age Z-score equal or below −2 ^2^weight for height Z-score equal or smaller than −2 ^3^for children 0–5 years defined as Z-score weight-for-age < −2; for children 5–18 years defined as Z-score BMI < −2. ^4^for children 0–5 years defined as Z-score weight-for-age > 2; for children 5–18 years defined as Z-score BMI > 1. *Percentage calculated for children under five.

Among the subgroup of UAMs, stunting was especially prevalent (31 %) as compared with 9 % among accompanied children (*p* < 0.01). Moreover, there was a difference in Z-scores of 0.86 for height-for-age between these two groups (*p* < 0.05).

We compared the nutritional status of children under two years old who were breastfed versus those who were not. Children who were breastfed demonstrated a non-significant higher WHZ (median Z-score for weight-for-height (Z-score difference of 1.4, *p* = 1.18). Noteworthy is that children in our study population were breastfed up to the age of four years old. Comparing children in this age group does give a statistically significant difference in Z-score between breastfed children, Z-score 0.44, vs non breastfed children, Z-score −0.38 (Z-score difference of 0.82, *p* < 0.01).

Of the accompanied children, 254 (90 %) were found to have the food distributed in camp as their main source of nutrition. The remainder reportedly consumed food from other sources. At the time of our study, the food was distributed once a day at 14:00 and included items such as pasta and rice.

A multivariable regression showed no significant associations between Z-score height-for-age at baseline and the following variables: number of siblings (β=0.06, 95 % CI −0.06–0.2, *p* = 0.3), length of stay in the camp (β=−0.08, 95 % CI −0.02–0.004, *p* = 0.2), and travel duration before camp arrival (β=0.1, 95 % CI −0.001–0.02, *p* = 0.7). There was also no significant difference in Z-score height-for-age at baseline and reported presence of chronic disease.

In terms of clinical signs of malnutrition, 36 (12 %) children showed excessive dental caries. Dry, scaly skin was observed in 15 (5 %) of the children. No children showed evidence of nutritional edema.

## Discussion

This study focuses on the nutritional status of children living in one of Europe’s largest refugee camps. The research aims to provide insight into the well-being of this vulnerable group of children.

We believe that it provides important tools to inform policies and raise awareness about their health. This gap in the literature may be in part due to the fact that research in a refugee camp—particularly among minors—involves navigating numerous practical challenges, including logistical difficulties, ethical considerations, and the fragile living conditions of a highly vulnerable pediatric population.

We found a prevalence of 11 % of stunting. 10 % of children under five were wasted. Of all children, 5 % underweight and 13 % were overweight. The percentage of stunted children is lower compared to the numbers reported in our meta-analysis among displaced children living in refugee camps in Europe and the MENA region, which showed a pooled prevalence of 16 % (Benjeddi et al., 2023). By contrast, we observed a higher rate of wasting of 10 %, compared to 4 % in the systematic review. One possible explanation is that our sample consists of children who have recently arrived at a European refugee camp, unlike the studies in our review, which involved children in Northern Greece or long-term refugee camps in the MENA region. The higher percentage of wasting may reflect the difficult and insecure food situation during the fleeing period.

The prevalence of overweight, mainly found in the older children, was an unexpected but concerning finding. This percentage exceeds WHO’s ‘high’ cut-off value, 10 %, of public health significance (de Onis et al., 2018). This prevalence could be explained by the double burden of malnutrition (Popkin et al., 2020). The percentage of overweight could be explained by *in utero* exposure to maternal undernutrition and relatively high levels of stunting earlier in life, that set the stage for later nutritional challenges such as overweight (Wells et al., 2020).,. This is in line with the Barker hypothesis and reflects the nutritional status of the mother (De Boo et al., 2006). . Not only does this impose risks for the individual in the short term, it also increases the risk of non-communicable diseases (e.g. diabetes type II, hypertension and stroke) later in life with all due consequences on a societal level (Nugent et al., 2020). . Furthermore, the large difference in the prevalence of stunting between UAMs and accompanied children is particularly noteworthy, further emphasizing the vulnerability of UAMs. This observed prevalence of stunting (31 %), exceeds the 'very high' cut-off value, 30 %, set by the WHO for public health significance (de Onis et al., 2018), underlining its alarming importance. Notably, UAMs are known to misreport their age, a behavior often driven by the wish for protective measures (Maioli et al., 2021). This tendency introduces a potential underestimation of the true amount of stunting in the researched group. The difference in stunting between accompanied and UAMs could be explained in different ways. Firstly, it could be hypothesized that UAMs have worse living conditions in the country of origin that make them travel alone in the first place. Secondly, they lack the protection of family which affects both their short- as well as long-term health, including food security. Thirdly, as the cause of stunting includes a myriad of factors - the explanation for this high level of stunting will likely involve decreased access to healthcare, inadequate nutrition, worse sanitation, and broader socioeconomic dimensions (Black et al., 2013).

Another subgroup analysis underscores the ongoing positive nutritional association with breastfeeding. Breastfeeding within refugee camps continues to be an insufficiently studied topic (Ebrahimi, 2021). Our observed higher mean Z-score for weight-for-height among breastfed children shows the protective effect of breastfeeding against undernutrition. This difference is non-significant and might be due to small sample size. Despite the circumstances of breastfeeding mothers, children might be better off in terms of their weight. This finding underscores the significance of promoting and supporting breastfeeding practices, especially in emergency settings like refugee camps.

The non-significant improvement of all malnutrition indicators over time indicates an improvement of nutritional status during their stay in camp. The only other prospective study on this topic involved Rohingya refugee children living in refugee camps in which nutritional status was assessed cross-sectionally twice (Leidman et al., 2020). This study found significant improvements in acute and micronutrient malnutrition among Rohingya children in makeshift settlements, likely due to the scale-up of nutritional services. An explanation could be that relative safety and stability have a positive impact on nutritional status. However, our findings regarding follow-up data were not statistically significant, which means that they should be interpreted with caution. While our measurements provide insights into the overall nutritional status, we lack information regarding micronutrient levels. It is possible that there is a net positive caloric balance resulting from the consumption of energy-dense but nutritionally unbalanced food provided by the food line. More thorough research is required to address this issue.

While our study has yielded very relevant findings, the following limitations should be taken into consideration. Firstly, the relatively small sample size in our study poses a constraint on the generalizability of our findings. Furthermore, the rapidly changing composition of the camp population means that our results mostly reflect the situation at the time of data-collection. A notable challenge encountered in our study was the substantial loss to follow-up which was inevitable due to the high mobility, namely families being moved from one refugee-camp to the other. Lastly, factors such as previous healthcare access, socioeconomic status, migration path, and availability of family support may influence both exposure and the outcomes observed. Due to the complexity of these factors and their interactions, they were beyond the scope of our study's objectives and were not included in our analysis.

**Fig. 2:**
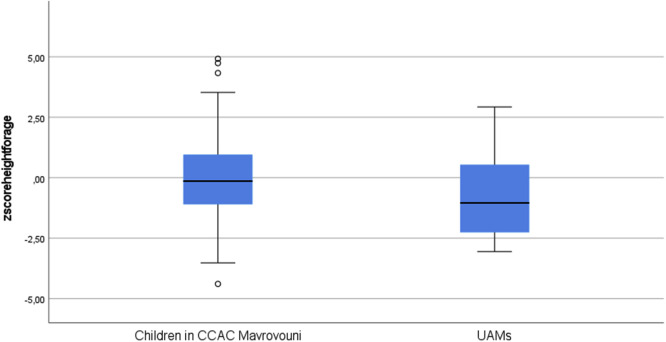
Boxplot reflecting the statistically significant difference in Z-score height for age between the group of accompanied and UAMs.

**Fig. 3:**
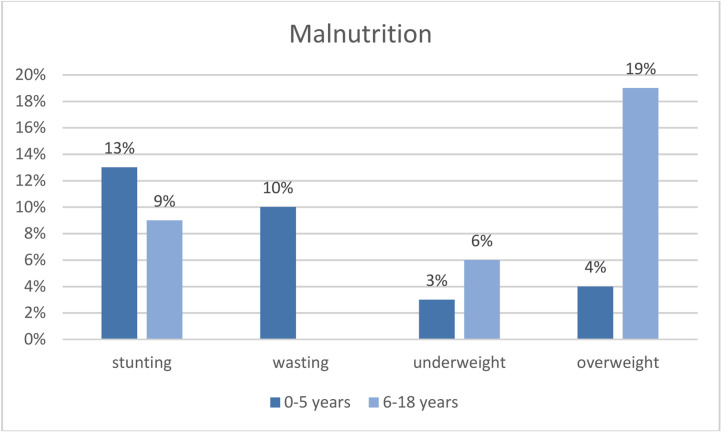
Percentages of malnutrition at baseline subdivided by age group.

It is vital for policymakers and humanitarian organizations to acknowledge that displaced children arriving in Europe often do so with a compromised nutritional status, adding an additional layer of vulnerability. UAMs represent a notably vulnerable subgroup, experiencing greater nutritional challenges and additionally having less support from a parental figure. Protecting the health of these children and ensuring access to adequate nutrition is crucial for their development Fig. 2, Fig. 3.

## Conclusion

In conclusion, this study represents an examination of the nutritional status of children in a European refugee camp, contributing valuable insights to an underexplored research field. The nutritional status of displaced children in camp Mavrovouni is compromised, with 11 % being stunted, 10 % wasted in children under five, and 13 % overweight overall The positive association between breastfeeding and weight-for-height Z-scores suggests that promoting breastfeeding can improve nutritional outcomes. This research not only contributes to the academic understanding of child health in refugee contexts but also provides actionable insights for policymakers, humanitarian organizations, and healthcare providers.

## Ethics committee approval

Ethical clearance for this research project was obtained from the Medical Ethics Committee of the Greek National Public Health Organization (EODY). This was provided in December 2022 under Reference No. 23,702/06–12–2022 (see Appendix II).

## Role of funding source

This study was funded by Stichting Weeshuis der Doopsgezinden and Stichting Steun Emma.

The funders of the study had no role in study design, data collection, data analysis, data interpretation, writing of the report or decision to publish the report.

## CRediT authorship contribution statement

**H. Benjeddi:** Writing – original draft, Visualization, Validation, Supervision, Software, Resources, Project administration, Methodology, Investigation, Funding acquisition, Formal analysis, Data curation, Conceptualization. **M.P. Gruppen:** Writing – review & editing, Supervision, Project administration, Data curation. **S.A. Post:** Data curation. **A.D. Groenewegen:** Data curation. **E.B.K. Egen:** Data curation. **E. Samiotaki Logotheti:** Data curation. **Z. Livaditou:** Supervision, Data curation. **W.P. Voskuijl:** Validation, Methodology, Formal analysis. **A.M. Tutu-van Furth:** Writing – review & editing, Supervision. **M. Boele van Hensbroek:** Writing – review & editing, Validation, Supervision, Funding acquisition, Formal analysis, Conceptualization. **A. Terzidis:** Supervision, Project administration, Conceptualization. **M. van der Kuip:** Writing – review & editing, Validation, Supervision, Formal analysis.

## Declaration of competing interest

The authors declare that they have no known competing financial interests or personal relationships that could have appeared to influence the work reported in this paper.

The author is an Editorial Board Member/Editor-in-Chief/Associate Editor/Guest Editor for this journal and was not involved in the editorial review or the decision to publish this article.

## Appendix Supplementary materials

Image, image 1

Image, application 2

Image, application 3
