# Supplementary figures and images for: Nutritional status of displaced children including unaccompanied minors on Lesvos, Greece
Source: J Migr Health. 2025 Dec 27;13:100393. doi: 10.1016/j.jmh.2025.100393 (PMC13404046; doi:10.1016/j.jmh.2025.100393)

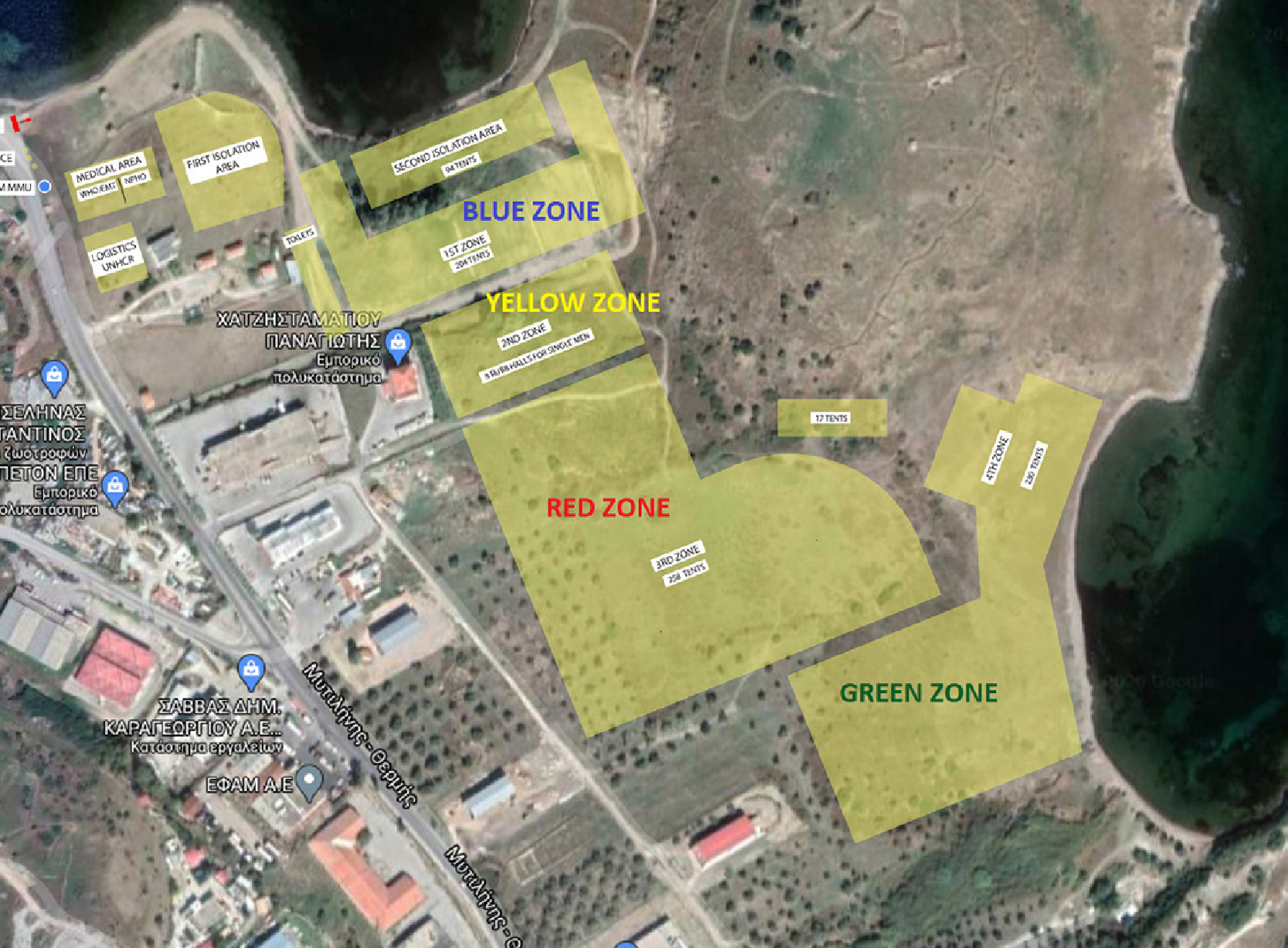

Supplement: Supplementary file 1 [file mmc1.jpg]
