# Supplementary material for: Nutritional status of displaced children including unaccompanied minors on Lesvos, Greece
Source: J Migr Health. 2025 Dec 27;13:100393. doi: 10.1016/j.jmh.2025.100393 (PMC13404046; doi:10.1016/j.jmh.2025.100393)
Supplement: Supplementary file 2 [file mmc2.pdf]

ΕΘΝΙΚΟΣ ΟΡΓΑΝΙΣΜΟΣ
ΔΗΜΟΣΙΑΣ ΥΓΕΙΑΣ

Ε.Ο.Δ.Υ.
ΚΠ 2018 / 2023 - 23/01/2023

Αγράφων 3-5, Μαρούσι
Τ.Κ. 15123
Τηλ. 2105212000
www.eody.gov.gr
: @eody
: @eody_gr

**Πληροφορίες:**
**Επιτροπή Ηθικής και Δεοντολογίας**
ΕΛΕΥΘΕΡΙΑ ΣΙΑΡΕ , 2105212040

**Προς :**

Κοσμήτορα Σχολής Επιστημών Υγείας,
Καθηγητή κ. Εμμανουήλ Πικουλή

Ιατρική Σχολή Πανεπιστημίου Αθηνών

**Θέμα:**Απάντηση στην υπ' αριθ. 23702/06-12-2022 αίτηση

Σε συνέχεια της υπ' αριθ. 23702/06-12-2022 αίτηση της κας HanaaBenjeddiπρος την Επιτροπή Ηθικής & Δεοντολογίας και κατόπιν του 2ου/16-12-2022 πρακτικού της Επιτροπής το οποίο εγκρίθηκε από το υπ' αριθ. 25298/23-12-2022 απόσπασμα πρακτικού της 7ης/22-12-2022 συνεδρίασης του ΔΣ του ΕΟΔΥ, σας ενημερώσουμε ότι έχει εγκριθεί.

**Ο Πρόεδρος ΔΣ ΕΟΔΥ**

**ΑΚΡΙΒΕΣ ΑΝΤΙΓΡΑΦΟ**
Προϊστάμενος Γραμματείας
α.α.
**Ναυσικά Παπαθανασίου**

ΕΛΛΗΝΙΚΗ ΔΗΜΟΚΡΑΤΙΑ ΥΠΟΥΡΓΕΙΟ ΥΓΕΙΑΣ ΕΟΔΥ

**Θεοκλής Ζαούτης**

**Εσωτερική Διανομή :**

Γραφείο Γραμματείας Προέδρου

Προϊστάμενος Διεύθυνσης Επιδημιολογικής Επιτήρησης & Παρέμβασης για Λοιμώδη Νοσήματα

Γραφείο Γραμματείας Αντιπροέδρου - Μεταδοτικά Νοσήματα

Γραφείο Γραμματείας Αντιπροέδρου - Μη Μεταδοτικά Νοσήματα

Αυτοτελές Τμήμα Διοίκησης

Προϊστάμενος Διεύθυνσης Επιδημιολογίας και Πρόληψης μη Μεταδοτικών Νοσημάτων και Τραυματισμών

Επιτροπή Ηθικής και Δεοντολογίας
