# Supplementary material for: Nutritional status of displaced children including unaccompanied minors on Lesvos, Greece
Source: J Migr Health. 2025 Dec 27;13:100393. doi: 10.1016/j.jmh.2025.100393 (PMC13404046; doi:10.1016/j.jmh.2025.100393)
Supplement: Supplementary file 3 [file mmc3.pdf]

Mavrovouni Migrant Child Health Project - version 19.91

Printed on 23-11-2023 14:14:02 by Hanaa Benjeddi

1. First visit - Demographics

Number	Question	Answers
1.1	Informed consent	◯ Yes<br>◯ No
1.2	Informed assent	◯ Yes<br>◯ No
1.3	Date of visit	[ ] [ ] [ ] *(dd-mm-yyyy)*
1.4	Date of birth	[ ] [ ] [ ] *(dd-mm-yyyy)*
1.5	Age	[ ]
1.6	Gender	◯ Female<br>◯ Male
1.7	Siblings (alive)	[ ]
1.8	Country of origin	◯ Afghanistan<br>◯ Albania<br>◯ Algeria<br>◯ Andorra<br>◯ Angola<br>◯ Antigua and Barbuda<br>◯ Argentina<br>◯ Armenia<br>◯ Australia<br>◯ Austria<br>◯ Azerbaijan<br>◯ Bahamas<br>◯ Bahrain<br>◯ Bangladesh<br>◯ Barbados<br>◯ Belarus<br>◯ Belgium<br>◯ Belize

- Benin
- Bhutan
- Bolivia
- Bosnia and Herzegovina
- Botswana
- Brazil
- Brunei
- Bulgaria
- Burkina Faso
- Burundi
- Cabo Verde
- Cambodia
- Cameroon
- Canada
- Central African Republic
- Chad
- Chile
- China
- Colombia
- Comoros
- Democratic Republic of the Congo
- Republic of the Congo
- Costa Rica
- Cote d Ivoire
- Croatia
- Cuba
- Cyprus
- Czech Republic
- Denmark
- Djibouti
- Dominica
- Dominican Republic
- Ecuador
- Egypt
- El Salvador
- Equatorial Guinea
- Eritrea
- Estonia
- Ethiopia
- Fiji
- Finland
- France
- Gabon
- Gambia
- Georgia
- Germany
- Ghana
- Greece

- Grenada
- Guatemala
- Guinea
- Guinea-Bissau
- Guyana
- Haiti
- Honduras
- Hungary
- Iceland
- India
- Indonesia
- Iran
- Iraq
- Ireland
- Israel
- Italy
- Jamaica
- Japan
- Jordan
- Kazakhstan
- Kenya
- Kiribati
- Kosovo
- Kuwait
- Kyrgyzstan
- Laos
- Latvia
- Lebanon
- Lesotho
- Liberia
- Libya
- Liechtenstein
- Lithuania
- Luxembourg
- Macedonia
- Madagascar
- Malawi
- Malaysia
- Maldives
- Mali
- Malta
- Marshall Islands
- Mauritania
- Mauritius
- Mexico
- Micronesia
- Moldova
- Monaco

- Mongolia
- Montenegro
- Morocco
- Mozambique
- Myanmar (Burma)
- Namibia
- Nauru
- Nepal
- Netherlands
- New Zealand
- Nicaragua
- Niger
- Nigeria
- North Korea
- Norway
- Oman
- Pakistan
- Palau
- Palestine
- Panama
- Papua New Guinea
- Paraguay
- Peru
- Philippines
- Poland
- Portugal
- Qatar
- Romania
- Russia
- Rwanda
- Saint Kitts and Nevis
- Saint Lucia
- Saint Vincent and the Grenadines
- Samoa
- San Marino
- Sao Tome and Principe
- Saudi Arabia
- Senegal
- Serbia
- Seychelles
- Sierra Leone
- Singapore
- Slovakia
- Slovenia
- Solomon Islands
- Somalia
- South Africa
- South Korea

- South Sudan
- Spain
- Sri Lanka
- Sudan
- Suriname
- Swaziland
- Sweden
- Switzerland
- Syria
- Taiwan
- Tajikistan
- Tanzania
- Thailand
- Timor-Leste
- Togo
- Tonga
- Trinidad and Tobago
- Tunisia
- Turkey
- Turkmenistan
- Tuvalu
- Uganda
- Ukraine
- United Arab Emirates
- United Kingdom
- United States of America
- Uruguay
- Uzbekistan
- Vanuatu
- Vatican City
- Venezuela
- Vietnam
- Yemen
- Zambia
- Zimbabwe

1.9	Months in camp	
1.10	Camp before	Yes<br>No
1.11	Months on the road	
1.12	Phone number for follow-up	
1.13	Current location Camp	

1.14	Is there any other relevant demographic information?	

2. First visit - Health information

Number	Question	Answers
2.1	Chronic disease	◯ Yes<br>◯ No
2.2	Chronic Disease	☐ Asthma<br>☐ Epilepsy<br>☐ Diabetes<br>☐ HIV<br>☐ Other, namely
2.3	Chronic disease specified	
2.4	Medication	
2.5	Vaccinated	◯ Yes<br>◯ No<br>◯ Unknown

3. First visit - Breastfeeding

Number	Question	Answers
3.1	Is the child currently being breastfed?	◯ Yes<br>◯ No

3.2	If breastfeeding, what is the mother's age?	
3.3	If not breastfeeding anymore, what was the duration of breastfeeding	☐ 0-3 mo<br>☐ 4-6 mo<br>☐ 7-12 mo<br>☐ > 12 mo<br>☐ Unknown<br>☐ N/A
3.4	In the country of origin, do new mothers breastfeed or prefer a different feeding option?	○ Prefer breastfeeding<br>○ Prefer formula
3.5	Answer the questions below only if mother is currently breastfeeding.	
3.6	Have you received any information about breastfeeding from the camp? If yes, from whom?	
3.7	Safety breastfeeding	☐ Yes<br>☐ No
3.8	Where do you breastfeed?	☐ At home (container, tent)<br>☐ A specific designed space for breastfeeding<br>☐ Other
3.9	Do you feel that you have an adequate diet to breastfeed?	☐ Yes<br>☐ No
3.10	Do you have enough water to breastfeed?	○ Yes<br>○ No

4. First visit - Diet

Number	Question	Answers
4.1	Food sources	☐ Foodline<br>☐ Bought from store or market<br>☐ Donations NGOs

		☐ Combination of above
4.2	What does the child eat on a typical day?	☐ Biscuits<br>☐ Breast Milk<br>☐ Cereals and Cereal Products<br>☐ Eggs<br>☐ Fats and Oils<br>☐ Fish and Sea Foods<br>☐ Fruits<br>☐ Meats<br>☐ Milk and Milk Products: Fresh/fermented milk, cheese, yogurt, or other milk products<br>☐ Pasta<br>☐ Poultry<br>☐ Pulses / Legumes / Nuts and Seeds<br>☐ Rice<br>☐ Roots<br>☐ Sugars / Honey and Commercial Juices<br>☐ Vegetables<br>☐ Miscellaneous<br>☐ Unknown
4.3	If breastfeeding, what does the mother eat on a typical day?	☐ Biscuits<br>☐ Breast Milk<br>☐ Cereals and Cereal Products<br>☐ Eggs<br>☐ Fats and Oils<br>☐ Fish and Sea Foods<br>☐ Fruits<br>☐ Meats<br>☐ Milk and Milk Products: Fresh/fermented milk, cheese, yogurt, or other milk products<br>☐ Pasta<br>☐ Poultry<br>☐ Pulses / Legumes / Nuts and Seeds<br>☐ Rice<br>☐ Roots<br>☐ Sugars / Honey and Commercial Juices<br>☐ Vegetables<br>☐ Miscellaneous<br>☐ Unknown
4.4	How does the child eat?	☐ Fed by adult<br>☐ Child feeds self, supervised by adult<br>☐ Child feeds self, supervised by older children<br>☐ Child feeds self, unsupervised

		☐ Fed from common plate or bowl<br>☐ Child exclusively breastfed
4.5	Number of meals	[ ]

5. First visit - Physical exam

Number	Question	Answers
5.1	Body weight	[ ] kg
5.2	Height	[ ] cm
5.3	MUAC	[ ] mm
5.4	Clinical malnutrition	☐ Oedema<br>☐ Pallor (pale)<br>☐ Dry, scale skin<br>☐ Dermatitis<br>☐ Pale or dull nails<br>☐ Enlarged thyroid<br>☐ Cracked lips<br>☐ Bleeding gums<br>☐ Xerophthalmia<br>☐ Dull/thin/sparse hair<br>☐ Excessive dental caries<br>☐ None

6. Follow-up 3 mo - Demographics 3 mo

Number	Question	Answers
6.1	Date of visit	[ ] [ ] [ ] *(dd-mm-yyyy)*

7. Follow-up 3 mo - Health 3 mo

Number	Question	Answers
7.1	Any health events in the past 3 months (since previous assessment (eg severe illness, admissions, AB courses, extra food supplementations received)?	

8. Follow-up 3 mo - Diet 3 mo

Number	Question	Answers
8.1	Food sources	☐ Foodline<br>☐ Bought from store or market<br>☐ Donations NGOs<br>☐ Combination of above
8.2	Diet	☐ Biscuits<br>☐ Breast Milk<br>☐ Cereals and Cereal Products<br>☐ Eggs<br>☐ Fats and Oils<br>☐ Fish and Sea Foods<br>☐ Fruits<br>☐ Meats<br>☐ Milk and Milk Products: Fresh/fermented milk, cheese, yogurt, or other milk products<br>☐ Pasta<br>☐ Poultry<br>☐ Pulses / Legumes / Nuts and Seeds<br>☐ Rice<br>☐ Roots<br>☐ Sugars / Honey and Commercial Juices<br>☐ Vegetables

		☐ Miscellaneous<br>☐ Unknown
8.3	Eating	☐ Fed by adult<br>☐ Child feeds self, supervised by adult<br>☐ Child feeds self, supervised by older children<br>☐ Child feeds self, unsupervised<br>☐ Fed from common plate or bowl<br>☐ Child exclusively breastfed
8.4	Number of meals	[ ]

9. Follow-up 3 mo - Physical examination 3 mo

Number	Question	Answers
9.1	Body weight	[ ] kg
9.2	Height	[ ] cm
9.3	MUAC	[ ] mm
9.4	Clinical malnutrition	☐ Oedema<br>☐ Pallor (pale)<br>☐ Dry, scale skin<br>☐ Dermatitis<br>☐ Pale or dull nails<br>☐ Enlarged thyroid<br>☐ Cracked lips<br>☐ Bleeding gums<br>☐ Xerophthalmia<br>☐ Dull/thin/sparse hair<br>☐ Excessive dental caries<br>☐ None

10. Follow-up 6 mo - Demographics 6 mo

Number	Question	Answers
10.1	Date of visit	☐ ☐ ☐ *(dd-mm-yyyy)*

11. Follow-up 6 mo - health 6 mo

Number	Question	Answers
11.1	Any health events in the past 3 months (since previous assessment (eg severe illness, admissions, AB courses, extra food supplementations received)?	☐

12. Follow-up 6 mo - Diet 6 mo

Number	Question	Answers
12.1	Food sources	☐ Foodline<br>☐ Bought from store or market<br>☐ Donations NGOs<br>☐ Combination of above
12.2	Diet	☐ Biscuits<br>☐ Breast Milk<br>☐ Cereals and Cereal Products<br>☐ Eggs<br>☐ Fats and Oils<br>☐ Fish and Sea Foods<br>☐ Fruits<br>☐ Meats

Number	Question	Answers
		☐ Milk and Milk Products: Fresh/fermented milk, cheese, yogurt, or other milk products<br>☐ Pasta<br>☐ Poultry<br>☐ Pulses / Legumes / Nuts and Seeds<br>☐ Rice<br>☐ Roots<br>☐ Sugars / Honey and Commercial Juices<br>☐ Vegetables<br>☐ Miscellaneous<br>☐ Unknown
12.3	Eating	☐ Fed by adult<br>☐ Child feeds self, supervised by adult<br>☐ Child feeds self, supervised by older children<br>☐ Child feeds self, unsupervised<br>☐ Fed from common plate or bowl<br>☐ Child exclusively breastfed
12.4	Number of meals	[ ]

13. Follow-up 6 mo - Physical examination 6 mo

Number	Question	Answers
13.1	Body weight	[ ] kg
13.2	Height	[ ] cm
13.3	MUAC	[ ] mm
13.4	Clinical malnutrition	☐ Oedema<br>☐ Pallor (pale)<br>☐ Dry, scale skin<br>☐ Dermatitis<br>☐ Pale or dull nails<br>☐ Enlarged thyroid<br>☐ Cracked lips<br>☐ Bleeding gums

- [ ] Xerophthalmia
- [ ] Dull/thin/sparse hair
- [ ] Excessive dental caries
- [ ] None
